# Effect of process mode, nitrogen source and temperature on L-malic acid production with *Aspergillus oryzae* DSM 1863 using acetate as carbon source

**DOI:** 10.3389/fbioe.2022.1033777

**Published:** 2022-10-14

**Authors:** Aline Kövilein, Lena Zadravec, Silja Hohmann, Julia Umpfenbach, Katrin Ochsenreither

**Affiliations:** Institute of Process Engineering in Life Sciences 2—Technical Biology, Karlsruhe Institute of Technology (KIT), Karlsruhe, Germany

**Keywords:** malate, succinate, organic acid, filamentous fungus, fed-batch, repeated-batch

## Abstract

Malic acid, mainly used as acidulant and taste enhancer in the food industry, is currently produced from fossil resources. In this study, microbial L-malate production with the filamentous fungus *A. oryzae* using the carbon source acetate was evaluated. Acetate is for example contained in biomass-derived substrates such as lignocellulosic hydrolysates and condensates of fast pyrolysis, thus avoiding competition with food production. Since research on malic acid synthesis from acetate is limited and reported productivities and yields are low, this work aimed to improve the process. First, different cultivation temperatures were tested. This parameter was found to affect the ratio between malic and succinic acid, which is the major by-product of organic acid production with *A. oryzae*. At 32°C, the malate share was highest (53.7 ± 1.6%), while it was lowest at 38°C (43.3 ± 1.1%) whereas succinate represented the main product (51.5 ± 1.0%). Besides the temperature, the type of nitrogen source was also found to affect malate synthesis as well as biomass production. In the pre-culture, the biomass concentration was increased by a factor of 3.4–3.9, and germination started earlier with the complex nitrogen sources yeast extract, casein hydrolysate and peptone compared to the defined nitrogen source (NH_4_)_2_SO_4_. Especially with yeast extract, malate synthesis in the main culture was accelerated and the titer obtained after 48 h was about 2.6 times higher than that quantified with (NH_4_)_2_SO_4_. To reduce substrate inhibition in acetate medium, fed-batch and repeated-batch processes were evaluated using (NH_4_)_2_SO_4_ or yeast extract as nitrogen source. In the fed-batch process, the period of malate production was extended, and the maximum product concentration was increased to 11.49 ± 1.84 g/L with (NH_4_)_2_SO_4_ and 12.08 ± 1.25 g/L with yeast extract. In the repeated-batch process, the total acid production was highest within the first 240 h of fermentation, but optimization is required to maintain high production rates in later cycles. The lessons learned in this study will help in the development of further process strategies to maximize malate production using acetate as alternative substrate to the commonly used glucose.

## Introduction

With fossil resources being of limited availability, the need for bio-based production of chemicals becomes increasingly important. However, processes using renewable raw materials are often not yet economic due to higher substrate costs and lower yields. Malic acid is one of the chemicals currently synthesized from fossil resources, although it has great potential to be produced microbially as an intermediate of the tricarboxylic acid cycle. Thus, various natural or metabolically engineered malic acid producers have been identified or designed which are capable of utilizing a variety of different substrates, including low-cost side or waste streams from the processing of renewable feedstock (Kövilein et al., 2020; Wei et al., 2021).

Acetate is a promising carbon source for the production of bio-based chemicals, as it is commonly found in substrates derived from biomass such as lignocellulosic hydrolysates or condensates of pyrolysis. Especially pyrolysis products can contain high concentrations of acetic acid in the range of 5%–17% (wt. dry basis) in bio-oil fractions (Bertero et al., 2012; Oh et al., 2017). In some processes, such as the fast pyrolysis performed during pretreatment of wheat straw at the bioliq^®^ pilot plant, an aqueous condensate is obtained besides the bio-oil fraction (Pfitzer et al., 2016). This side stream, characterized by a low heating value, usually contains 30–50 g/L acetic acid (Arnold et al., 2019; Kubisch and Ochsenreither, 2022), which was demonstrated to be a suitable concentration range for L-malic acid production with *A. oryzae* (Kövilein et al., 2021). Although *A. oryzae*, which is a potent natural producer of L-malic acid, can use acetate to produce the dicarboxylic acid, maximum concentrations and productivities are currently much lower compared to processes using glucose. In batch fermentations, the maximum malic acid titer obtained with acetate as sole carbon source was below 10 g/L when an optimal substrate concentration of 45 g/L was applied (Kövilein et al., 2021). In contrast, when glucose was used, concentrations of 150–200 g/L were achieved with *Aspergillus* species (Liu et al., 2017; Xu et al., 2020; Ji et al., 2021). However, these high concentrations were obtained with genetically modified strains in fed-batch processes. A batch process is especially unfavorable when acetate is the carbon source due to substrate inhibition observed at rather low concentrations. Further challenges which accompany the microbial utilization of this substrate are a high ion concentration in acetate-rich media which can affect the morphology (Kövilein et al., 2021), and a pH-dependent toxicity. The inhibitory mechanism of acetate is assumed to be mainly caused by its protonated form which can diffuse into the cytoplasm where it deprotonates due to the near-neutral pH, causing acidification (Stratford et al., 2009). Thus, the pH-range suitable for fermentation with this carbon source is limited. These characteristics add to the complexity of microbial product synthesis, which is why acetate has received less attention than sugars such as glucose, despite its large potential as lignocellulose-derived substrate in a bioeconomy (Kiefer et al., 2021). For efficient product synthesis from acetate, processes need to be adapted to these characteristics. An advantage of acetate utilization is the possibility to counteract the pH increase caused by acetate consumption through the feeding of additional substrate in the form of acetic acid. Therefore, acetate-based production of malic acid in fed-batch mode is likely to enhance the fermentation outcome by avoiding high initial substrate concentrations and maintaining the pH-value in a defined range. A repeated-batch process furthermore prevents the occurrence of high product titers through the periodic exchange of medium, thus minimizing product inhibition. With glucose as substrate, a repeated-batch process has already been shown to extend the production time and enhance malic acid productivity with *A. oryzae* (Schmitt et al., 2021).

Besides the process mode, the cultivation temperature and type of nitrogen source can also have a significant impact on microbial production. Some nitrogen sources have already been evaluated for L-malic acid synthesis with *A. oryzae*, but with different strains and glucose as substrate. In these studies, the complex nitrogen sources peptone and tryptone were found to result in considerably higher malic acid concentrations than ammonium compounds (Knuf et al., 2013; Ding et al., 2018). The optimum cultivation temperature varies depending on the microorganism and target product. Using *A. oryzae* DSM 1863 and acetate as carbon source, the effects of temperature on organic acid synthesis have not yet been studied. For the production of succinic, itaconic or malic acid with other *Aspergillus* species, temperatures between 30 and 35°C were reported most suitable (Chen et al., 2019; Saha et al., 2019; Yang et al., 2020).

This study aimed to evaluate strategies for improving L-malic acid production with *A. oryzae* DSM 1863 using acetate as carbon source. First, organic acid production was investigated at cultivation temperatures in the range of 29–38°C and the side product spectrum was analyzed. Furthermore, different defined and complex nitrogen sources were evaluated for growth and organic acid production. Eventually, batch, fed-batch and repeated-batch fermentations were compared with the aim to increase the maximum malic acid concentration and enhance the productivity.

## Materials and methods

### Microorganism and media

*A. oryzae* DSM 1863 was obtained from DSMZ strain collection (German Collection of Microorganisms and Cell Cultures GmbH) and spore propagation was performed as described previously (Kövilein et al., 2022).

The basis pre-culture medium was composed of 45 g/L acetic acid, 0.75 g/L KH_2_PO_4_, 0.98 g/L K_2_HPO_4_, 0.1 g/L MgSO_4_·7H_2_O, 0.1 g/L CaCl_2_·2H_2_O, 5 mg/L NaCl, and 5 mg/L FeSO_4_·7H_2_O. The basis main culture medium for organic acid production consisted of 45 g/L acetic acid, 0.1 g/L KH_2_PO_4_, 0.17 g/L K_2_HPO_4_, 0.1 g/L MgSO_4_·7H_2_O, 0.1 g/L CaCl_2_·2H_2_O, 5 mg/L NaCl, 60 mg/L FeSO_4_·7H_2_O, and 90 g/L CaCO_3_. The nitrogen source and further modifications of these media specific for each experiment are indicated in the following sections. The pH of all pre-culture media containing acetate was set to 6.5 with NaOH. For the main culture media containing acetate, the pH was adjusted to a value of 5.5 with NaOH.

The experiments comparing different cultivations temperatures and the effect of Hutner’s trace elements solution with acetate as carbon source were compared to cultivations using glucose. Instead of acetic acid, the basis pre- and main culture medium then contained 40 g/L (pre-culture) or 120 g/L (main culture) glucose monohydrate. The pH of the glucose media was not adjusted.

Hutner’s trace element solution (1000×) was added to all pre-culture media except for those used in the experiments comparing different nitrogen sources. This solution consists of 5 g/L FeSO_4_·7H_2_O, 50 g/L EDTA-Na_2_, 22 g/L ZnSO_4_·7H_2_O, 11 g/L H_3_BO_3_, 5 g/L MnCl_2_·4H_2_O, 1.6 g/L CoCl_2_·6H_2_O, 1.6 g/L CuSO_4_·5H_2_O, and 1.1 g/L (NH_4_)_6_Mo_7_O_24_·4H_2_O with a pH of 6.5 (Hill and Kafer, 2001).

All pre- and main culture media were sterilized by autoclaving for 20 min at 121°C.

### Cultivation conditions

The following describes the general cultivation conditions that apply to all experiments presented in this study. Details regarding the conditions for individual experiments are given in the following sections.

100 ml of pre-culture medium in 500 ml baffled shake flasks was inoculated with 3 × 10^7^ conidia and incubated at 30°C and 100 rpm. After 48 h (media containing acetate) or 24 h (media containing glucose) of incubation, the biomass was separated from the medium by filtration through sterile Miracloth (Merck KGaA, Darmstadt, Germany), which had been autoclaved for 20 min at 121°C, and washed thoroughly with distilled water.

Organic acid production was performed in 500 ml baffled shake flasks containing 100 ml main culture medium. In the experiments comparing different nitrogen sources, 0.75 g of washed biomass was used for the inoculation of one main culture flask. The biomass was weighed with a balance under sterile conditions in a laminar flow hood after filtration through sterile Miracloth. For all other experiments, the biomass of two pre-cultures was resuspended in 200 ml main culture medium and 10 ml were used for the inoculation of one shake flask. The main culture was incubated at 120 rpm and 32°C unless stated otherwise.

### Evaluation of temperature effects on organic acid production

Organic acid production was evaluated at 29, 32, 35 and 38°C. In addition to acetate, experiments were also run with glucose as carbon source. For the main cultures with acetate, pre-cultures grown on acetate were used, while for the main cultures containing glucose, biomass formed with glucose was used. The pre-cultures with both carbon sources were grown with 4.00 g/L (NH_4_)_2_SO_4_ and 2 ml/L Hutner’s trace elements solution. All temperatures were tested as biological quadruplicates.

### Evaluation of nitrogen sources for growth and organic acid production

The concentration of all nitrogen sources added to the basis pre- and main culture media was equivalent to the nitrogen concentration of 4.00 g/L (NH_4_)_2_SO_4_ (pre-culture) or 1.20 g/L (NH_4_)_2_SO_4_ (main culture). This refers to a nitrogen concentration of about 0.85 g/L in the pre-culture and 0.25 g/L the main culture. For the complex nitrogen sources, the total nitrogen concentration as indicated by the manufacturer was used for calculating their respective amounts. The following concentrations were therefore applied in the pre-culture: 4.00 g/L (NH_4_)_2_SO_4_, 5.15 g/L NaNO_3_, 1.82 g/L urea, 12.31 g/L l-glutamic acid monopotassium salt monohydrate, 8.00 g/L yeast extract, 10.87 g/L casein hydrolysate, and 6.95 g/L peptone from soy. In the main culture the concentrations were the following: 1.20 g/L (NH_4_)_2_SO_4_, 0.55 g/L urea, 3.69 g/L l-glutamic acid monopotassium salt monohydrate, 2.40 g/L yeast extract, 3.26 g/L casein hydrolysate, and 2.09 g/L peptone from soy. The total nitrogen (TN) and amino nitrogen (AN) values for the complex N-sources were the following: yeast extract (10.6% TN, 5.4% AN), casein hydrolysate (acid-hydrolyzed, 7.8% TN, 5.8% AN), peptone from soy (papainic digested, 12.2% TN, 3.7% AN). All nitrogen compounds were purchased from Carl Roth GmbH + Co. KG except for l-glutamic acid monopotassium salt monohydrate which was obtained from Alfa Aesar. Urea was not autoclaved with the medium but added in the form of a sterile filtered stock solution afterwards. The biomass used for the inoculation of all main culture conditions was grown in the basis pre-culture medium containing 4.00 g/L (NH_4_)_2_SO_4_. All experiments for evaluating the effect of the nitrogen source in pre- or main culture were performed as biological triplicates.

### Evaluation of process modes

In 500 ml baffled shake flasks the batch process was compared to a fed-batch and repeated-batch process using acetate as carbon source. Each process mode was tested with either 1.20 g/L (NH_4_)_2_SO_4_ or 2.40 g/L yeast extract as nitrogen source in the main culture while all pre-cultures were grown using the basis medium containing 4.00 g/L (NH_4_)_2_SO_4_ and 2 ml/L Hutner’s solution. The yeast extract was the same as in the experiments evaluating different nitrogen sources.

In the fed-batch process, the acetic acid concentration in each shake flask was measured every 48 h, and 5 M acetic acid was added to obtain a concentration of about 45 g/L acetic acid. Hence, “fed-batch” in this publication refers to a process with feeds of acetic acid only.

In the repeated-batch process, the medium was first exchanged after 96 h and then every 72 h. Thereby, the biomass was filtered through Miracloth, washed with distilled water, and transferred to a new flask containing 100 ml of fresh main culture medium, including 90 g/L CaCO_3_. Hence, the term “repeated-batch” in this work refers to a process with complete medium replacement and reutilization of the entire amount of biomass in each cycle.

All experiments for the evaluation of process modes were performed as biological triplicates.

### Analytics

For the determination of the biomass concentration at the end of the pre-culture, pre-weighed Miracloth was used for the filtration of the entire content of one flask. The biomass-containing Miracloth was dried at 70°C until the weight remained constant. Afterwards, the weight of the Miracloth with the biomass was measured with precision scales to calculate the dry biomass concentration.

Organic acid and glucose concentration was quantified by HPLC (Agilent 1100 Series) using a Rezex ROA organic acid H+ (8%) column (300 by 7.8 mm, 8 μm, Phenomenex) with a UV detector at 220 nm for organic acid and an RI detector for glucose determination. Conditions for analysis and sample preparation are described elsewhere (Kövilein et al., 2022).

Ammonium was measured photometrically using a Spectroquant test kit (114752, Merck KGaA). The reaction volume of the assay was scaled down to 200 μl and samples were measured in duplicate in microtiter plates according to the instructions of the manufacturer.

## Results

### Effect of temperature on organic acid production

Four cultivation temperatures (29, 32, 35 and 38°C) were tested for organic acid production with *A. oryzae*. In addition to cultures with acetate as sole carbon source, the temperature effects were also investigated using glucose. Differences in organic acid productivity and composition of the acid spectrum as a function of cultivation temperature were observed for both substrates.

**FIGURE 1:**
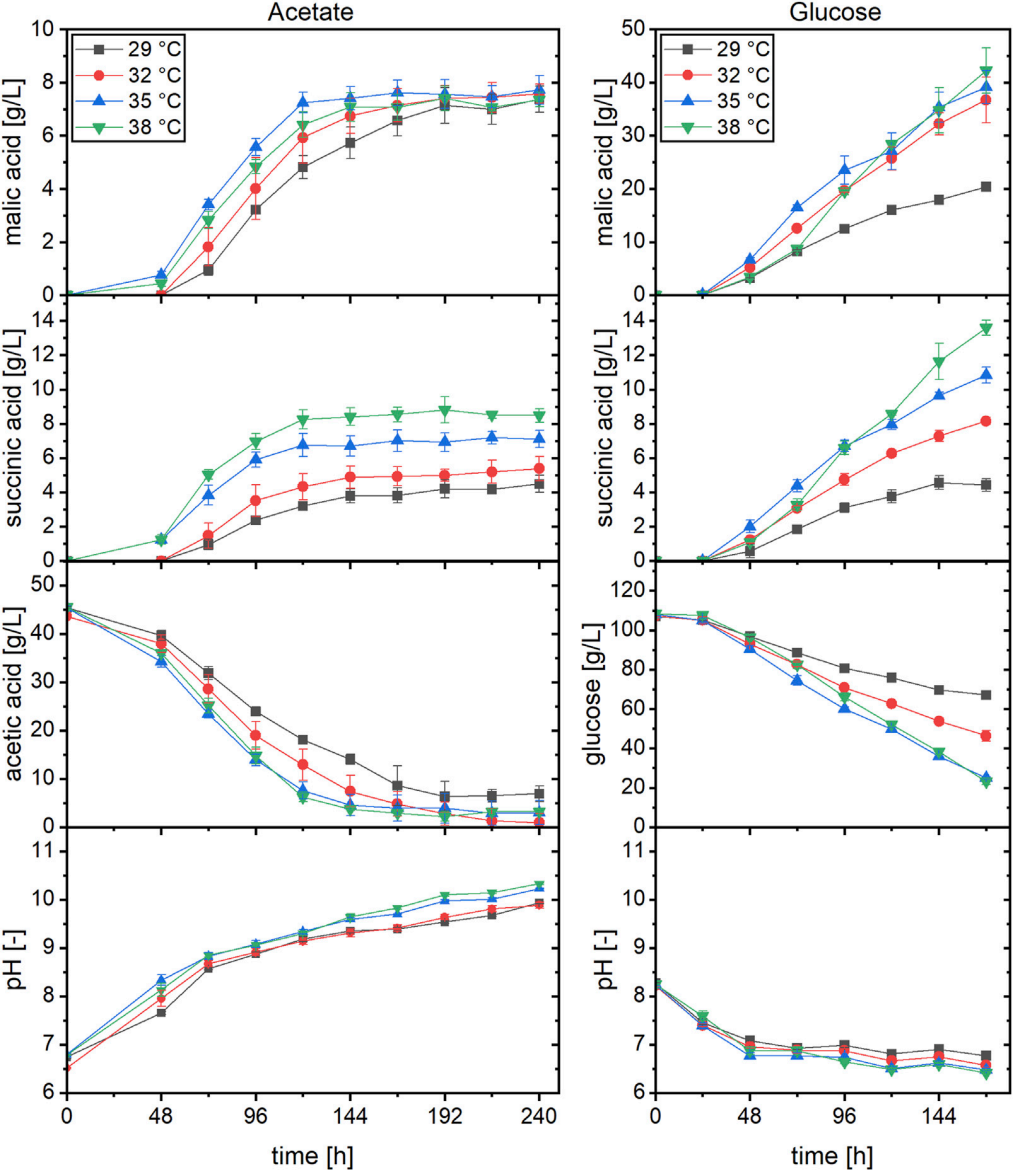
Malic and succinic acid production at different cultivation temperatures with acetate or glucose as carbon source. Datapoints represent means ± standard deviation, *n* = 4.

**FIGURE 2:**
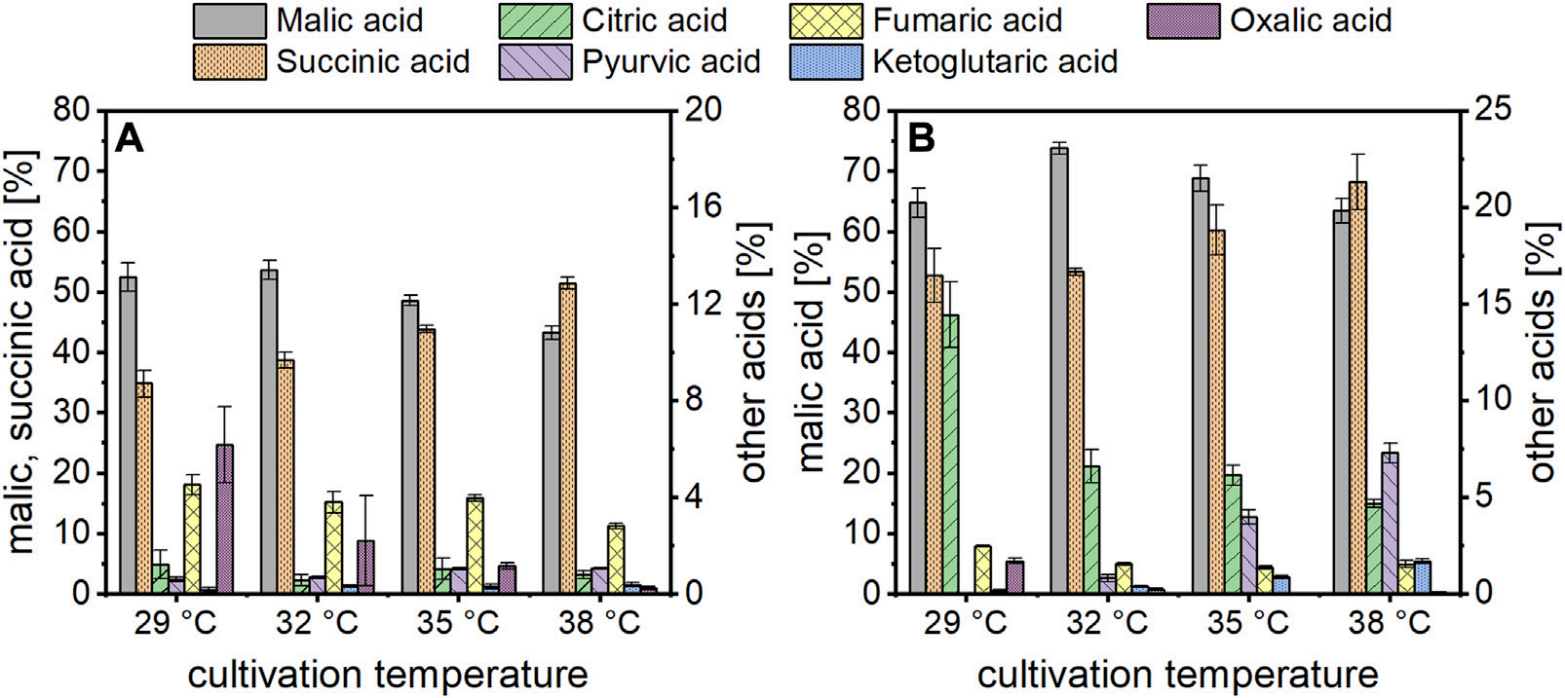
Organic acid composition obtained after 144 h of cultivation at different temperatures with acetate **(A)** or glucose **(B)** as carbon source. In **(A)** the left axis covers the malate and succinate percentages, while the right axis displays the data for the other products. In **(B)**, the left axis presents only the malic acid percentages, while the right axis covers all side products, including succinate. Datapoints represent means ± standard deviation, *n* = 4.

In the cultures with acetate, organic acid production started earlier when incubated at 35 or 38°C compared to the two lower temperatures (Figure 1). Regarding the production of malic acid, a cultivation temperature of 35°C resulted in the fastest product synthesis. At this temperature, production was already finished after about 120 h while an increase in malic acid concentration was detected until 192 h in cultures incubated at 29°C. Despite the differences in production velocity, the malic acid concentration obtained after 240 h of cultivation was similar for all temperatures. This was different for succinic acid production, which was the major by-product. The succinic acid concentration after 48 h was similar at 35 and 38°C (about 1.3 g/L) while no succinate was detected in cultures incubated at the lower temperatures at this time. From 72 h, the succinate concentration was proportional to the cultivation temperature, with the highest value obtained at 38°C. This concentration difference was observed until the end of the cultivation and resulted in a higher succinic than malic acid production in cultures incubated at 38°C. The substrate was almost completely consumed in cultures incubated at 32–38°C, whereas about 6.5 g/L acetic acid remained after 240 h of cultivation at 29°C (Figure 1). Since the consumption of acetate is accompanied by an alkalinization of the medium, the pH increased to values of about 9.9–10.3 after 240 h (Figure 1). Table 1 summarizes the cultivation outcomes after 144 h. At this sampling point, malic acid production was completed in most cultures. The lowest acetate consumption and substrate production was observed in cultures incubated at 29°C, with a total acid concentration of 10.91 ± 0.77 g/L. In cultures incubated at 38°C, the total acid concentration was highest (16.36 ± 1.03 g/L) but about half of this was succinic acid. Besides malate and succinate, also citric, pyruvic, fumaric, α-ketoglutaric and oxalic acid were detected. Figure 2 shows the percentages of these products related to the total acid concentration as given in Table 1. In Figure 2A, the left axis displays the main products malate and succinate while the right axis covers the remaining acids. The cultivation temperature affected the oxalate concentration considerably, as 6.2 ± 1.6% of this acid were produced in cultures incubated at 29°C compared to 0.3 ± 0.1% at 38°C. Furthermore, the pyruvate and ketoglutarate percentages showed a slight increase and the fumarate percentage a slight decrease with increasing temperature. For citrate, no temperature dependent trend was found with acetate as carbon source. The numerical values which are displayed in Figure 2 can be found in Supplementary Table S1.

**TABLE 1:** Fermentation results for cultivations of *A. oryzae* at different temperatures after 144 h. Values are means ± standard deviation, *n* = 4.

Substrate	T [°C]	Substrate metabolized [g/L]	Malic acid [g/L]	Y_P/S, malic acid_ [g/g]^a^	Malic acid productivity [g/L/h]	Succinic acid [g/L]	Total acid [g/L]^b^	Y_P/S, total acid_ [g/g]	Total acid productivity [g/L/h]
Acetate	29	31.32 ± 1.17	5.73 ± 0.59	0.18 ± 0.01	0.040 ± 0.004	3.80 ± 0.41	10.91 ± 0.77	0.35 ± 0.01	0.076 ± 0.005
	32	36.25 ± 3.53	6.74 ± 0.67	0.19 ± 0.00	0.047 ± 0.005	4.88 ± 0.65	12.60 ± 1.55	0.35 ± 0.02	0.087 ± 0.011
	35	40.90 ± 2.11	7.41 ± 0.44	0.18 ± 0.00	0.051 ± 0.003	6.70 ± 0.60	15.25 ± 1.17	0.37 ± 0.01	0.106 ± 0.008
	38	41.79 ± 0.34	7.08 ± 0.54	0.17 ± 0.01	0.049 ± 0.004	8.42 ± 0.52	16.36 ± 1.03	0.39 ± 0.03	0.114 ± 0.007
Glucose	29	37.46 ± 0.57	17.95 ± 0.68	0.48 ± 0.01	0.125 ± 0.005	4.57 ± 0.41	27.73 ± 0.48	0.74 ± 0.02	0.193 ± 0.003
	32	53.47 ± 1.73	32.25 ± 2.07	0.60 ± 0.06	0.224 ± 0.014	7.28 ± 0.33	43.71 ± 2.20	0.82 ± 0.06	0.304 ± 0.015
	35	72.02 ± 0.94	35.32 ± 2.83	0.49 ± 0.04	0.245 ± 0.020	9.63 ± 0.20	51.29 ± 2.60	0.71 ± 0.04	0.356 ± 0.018
	38	70.10 ± 0.95	34.78 ± 4.30	0.50 ± 0.05	0.242 ± 0.030	11.64 ± 1.06	54.76 ± 5.57	0.78 ± 0.07	0.380 ± 0.039

^a^ Y_P/S, malic acid_ [g/g] = substrate specific malic acid yield calculated as g (malic acid)/g (consumed substrate).

^b^ Refers to all organic acids quantified (see Figure 2).

The same temperatures were also evaluated in cultivations using glucose. With this carbon source as well, malic acid production was fastest at 35°C in the beginning which was surpassed by the cultures incubated at 38°C (Figure 1). Different than with acetate, the malic acid concentration of the cultures incubated at 29°C did not level up to the three higher temperatures in later stages of fermentation. Regarding the succinate concentration, on the other hand, similar observations were made as for acetate, with the highest concentration found at 38°C and the lowest at 29°C. The substrate consumption matched these observations, and the highest carbon utilization was quantified for cultures incubated at 35 and 38°C (Table 1; Figure 1). Except for cultures incubated at 29°C, malic acid concentration after 144 h was above 30 g/L, and the highest total acid concentration of 54.76 ± 5.57 g/L was obtained at a temperature of 38°C. Due to the buffering capacity of the added CaCO_3_, the pH was rather stable at values of about 6.5–7.0 after an initial decline (Figure 1). The temperature also affected the organic acid distribution, largely in a similar manner to the findings described for acetate. In Figure 2B, the left axis displays the malic acid percentages while the right axis covers the remaining acids (succinate, citrate, pyruvate, fumarate, α-ketoglutarate and oxalate). The oxalic and fumaric acid percentages decreased while the succinic, α-ketoglutaric, and pyruvic acid percentages increased with increasing temperature. Different than with acetate, the proportion of citric acid was affected by the cultivation temperature, decreasing from 14.4 ± 1.7% at 29°C to 4.7 ± 0.2% at 38°C.

For both substrates, malic acid yield was highest in cultures incubated at 32°C, amounting to 0.19 ± 0.00 g/g with acetate and 0.60 ± 0.06 g/g with glucose (Table 1). Hence, the relation of the product to the side products was most suitable for malic acid production at this cultivation temperature which was therefore used in the following experiments.

### Effect of nitrogen source on growth of *A. oryzae*

Growth of *A. oryzae* was evaluated with different defined and complex nitrogen sources using acetate as substrate. The nitrogen concentration in these experiments was adjusted to 0.85 g/L in all media. Regarding the defined nitrogen sources, biomass production was similar with (NH_4_)_2_SO_4_, urea and glutamate (Table 2). In these cultures, a dry biomass concentration of about 0.9 g/L was determined after 48 h of growth, whereas the lowest concentration of 0.48 ± 0.06 g/L was obtained with NaNO_3_. The utilization of complex nitrogen sources considerably enhanced the growth of *A. oryzae*. While similar dry biomass concentrations were determined with yeast extract and peptone (3.59 ± 0.14 and 3.58 ± 0.05 g/L, respectively), a slightly lower value of 3.11 ± 0.08 g/L was obtained with casein hydrolysate. The biomass yield was lowest for cultures containing NaNO_3_ (0.12 ± 0.01 g/g), followed by (NH_4_)_2_SO_4_ and urea (Table 2). Yields with the complex nitrogen sources were considerably higher, with the highest value of 0.79 ± 0.05 g/g obtained with peptone.

**TABLE 2:** Comparison of growth of *A. oryzae* after 48 h of cultivation in pre-culture medium with different nitrogen sources. Values are means ± standard deviation, *n* = 3.

Nitrogen source	Acetate metabolized [g/L]	Dry biomass [g/L]	Y_X/S_ [g/g]
(NH_4_)_2_SO_4_	5.63 ± 0.20	0.92 ± 0.13	0.16 ± 0.02
(NH_4_)_2_SO_4_ + 2 ml/L Hutner’s solution	5.59 ± 0.40	3.10 ± 0.25	0.56 ± 0.04
NaNO_3_	4.11 ± 0.09	0.48 ± 0.06	0.12 ± 0.01
Urea	5.18 ± 0.57	0.90 ± 0.23	0.17 ± 0.02
Glutamate	4.47 ± 0.24	0.92 ± 0.03	0.25 ± 0.06
Yeast extract	7.83 ± 0.90	3.59 ± 0.14	0.46 ± 0.05
Casein hydrolysate	7.65 ± 0.26	3.11 ± 0.08	0.41 ± 0.02
Peptone	4.47 ± 0.29	3.58 ± 0.05	0.79 ± 0.05

The nitrogen source also affected the onset of germination. When NaNO_3_ was used, growth was delayed as germination was only detected after 24 h of cultivation (Supplementary Figure S1A). With the other defined nitrogen sources urea, glutamate and (NH_4_)_2_SO_4_, biomass formation was already observed at this time point. This is exemplarily shown for cultures with (NH_4_)_2_SO_4_ in Supplementary Figure S1A. With all complex nitrogen sources germination was accelerated, as exemplarily shown for yeast extract in Supplementary Figure S1A. In these cultures, the majority of conidia had already started to form germ tubes after 8 h, whereas this was only sporadically observed with (NH_4_)_2_SO_4_ at this point.

The addition of Hutner’s trace element solution to cultures with (NH_4_)_2_SO_4_ also considerably enhanced biomass production, resulting in a concentration of 3.10 ± 0.25 g/L, which is a 3.4-fold increase compared to the cultures without trace elements. Thus, a similar dry biomass concentration was obtained as with casein hydrolysate, and the second highest biomass yield of 0.56 ± 0.04 g/g was determined. The trace element addition resulted in the formation of shorter biomass branches but did not accelerate the onset of germination (Supplementary Figure S1A). The effect of the trace element solution was also studied with glucose as carbon source, and similar observations were made. The addition of 2 ml/L of the trace element solution increased the dry biomass concentration by a factor of 2.9 (Supplementary Figure S2) and shorter and more branched filaments were formed (Supplementary Figure S1B). Increasing the trace element solution’s concentration to 4 or 6 ml/L had no significant effect on biomass production for either carbon source (Supplementary Figure S2).

### Effect of nitrogen source on L-malic acid production

**FIGURE 3:**
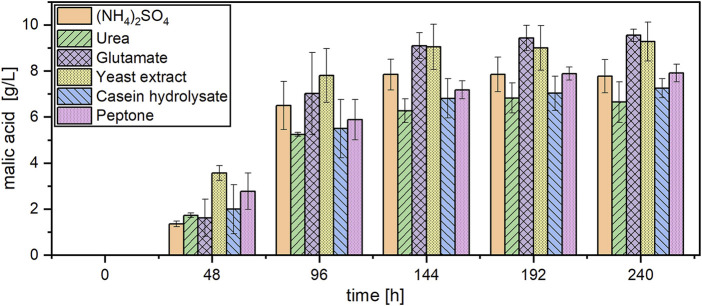
Malic acid production with *A. oryzae* using different nitrogen sources. Datapoints represent means ± standard deviation, *n* = 3.

The effect of different nitrogen sources was also evaluated during organic acid production. Due to the poor results obtained with NaNO_3_ in the pre-culture, this nitrogen source was not further considered for acid production. The course of malic acid concentration with different nitrogen sources is shown in Figure 3. Malic acid was detected in all cultures after 48 h. However, yeast extract considerably accelerated organic acid production especially in the beginning of the fermentation. Within the first 48 h, the highest malic acid concentration of 3.56 ± 0.32 g/L was detected with this nitrogen source, while the cultures with ammonium sulfate produced only 1.36 ± 0.13 g/L within the same time. The steepest concentration increase for all cultures was observed between 48 and 96 h. After 144 h of cultivation, only minor changes in acid concentration were observed, for which reason the calculations in Table 3 are based on this measurement point. After 144 h, the highest malic acid concentrations were quantified in cultures with glutamate (9.10 ± 0.56 g/L) and yeast extract (9.05 ± 0.98 g/L), resulting in a volumetric productivity of 0.063 g/L/h. The lowest acid production of 6.27 ± 0.51 g/L was detected in cultures with urea which also resulted in the lowest yield of 0.16 ± 0.00 g/g. The total acid concentration followed the same pattern, with the highest concentrations obtained with yeast extract and glutamate. With peptone, the lowest substrate consumption was observed with about 10 g/L acetic acid remaining after 144 h. In these cultures, 7.89 ± 0.24 g/L acetate was still detected at the end of the cultivation after 240 h while in most other cultures the carbon source was depleted or close to depletion at this time (Supplementary Figure S3). The pH development showed only little differences between the nitrogen sources, with all cultures starting at a value of about 6.5 and increasing to pH 9.8–10.0 after 240 h (Supplementary Figure S3).

**TABLE 3:** Fermentation results for cultivations of *A. oryzae* with different nitrogen sources after 144 h. Values are means ± standard deviation, *n* = 3.

Nitrogen source	Substrate metabolized [g/L]	Malic acid [g/L]	Y_P/S, malic acid_ [g/g]^a^	Malic acid productivity [g/L/h]	Total acid [g/L]^b^	Y_P/S, total acid_ [g/g]	Total acid productivity [g/L/h]
(NH_4_)_2_SO_4_	43.31 ± 0.72	7.84 ± 0.67	0.18 ± 0.01	0.054 ± 0.005	14.30 ± 0.85	0.33 ± 0.02	0.099 ± 0.006
Urea	38.11 ± 2.75	6.27 ± 0.51	0.16 ± 0.00	0.044 ± 0.004	12.74 ± 1.22	0.33 ± 0.01	0.088 ± 0.009
Glutamate	41.76 ± 3.28	9.10 ± 0.56	0.22 ± 0.00	0.063 ± 0.004	16.89 ± 1.30	0.40 ± 0.01	0.117 ± 0.009
Yeast extract	42.84 ± 1.87	9.05 ± 0.98	0.21 ± 0.01	0.063 ± 0.007	17.21 ± 1.51	0.40 ± 0.02	0.120 ± 0.010
Casein hydrolysate	39.16 ± 4.12	6.81 ± 0.86	0.17 ± 0.00	0.047 ± 0.006	13.09 ± 1.44	0.33 ± 0.00	0.091 ± 0.010
Peptone	34.11 ± 0.65	7.18 ± 0.39	0.21 ± 0.01	0.050 ± 0.003	14.16 ± 0.67	0.42 ± 0.02	0.098 ± 0.005

^a^ Y_P/S, malic acid_ [g/g] = substrate specific malic acid yield calculated as g (malic acid)/g (consumed acetic acid).

^b^ Refers to all organic acids quantified (see Supplementary Table S2).

Regarding the total acid composition, the malate share was lowest (49.3 ± 1.5%) and the oxalate percentage was highest (4.5 ± 1.2%) with urea, while malate was highest (54.8 ± 2.3%) and oxalate lowest (1.8 ± 0.4%) in the cultures with ammonium sulfate (Supplementary Table S2). Overall, however, the type of nitrogen source does not appear to have a considerable effect on the proportions of by-products.

### Comparison of process modes for L-malic acid production

**FIGURE 4:**
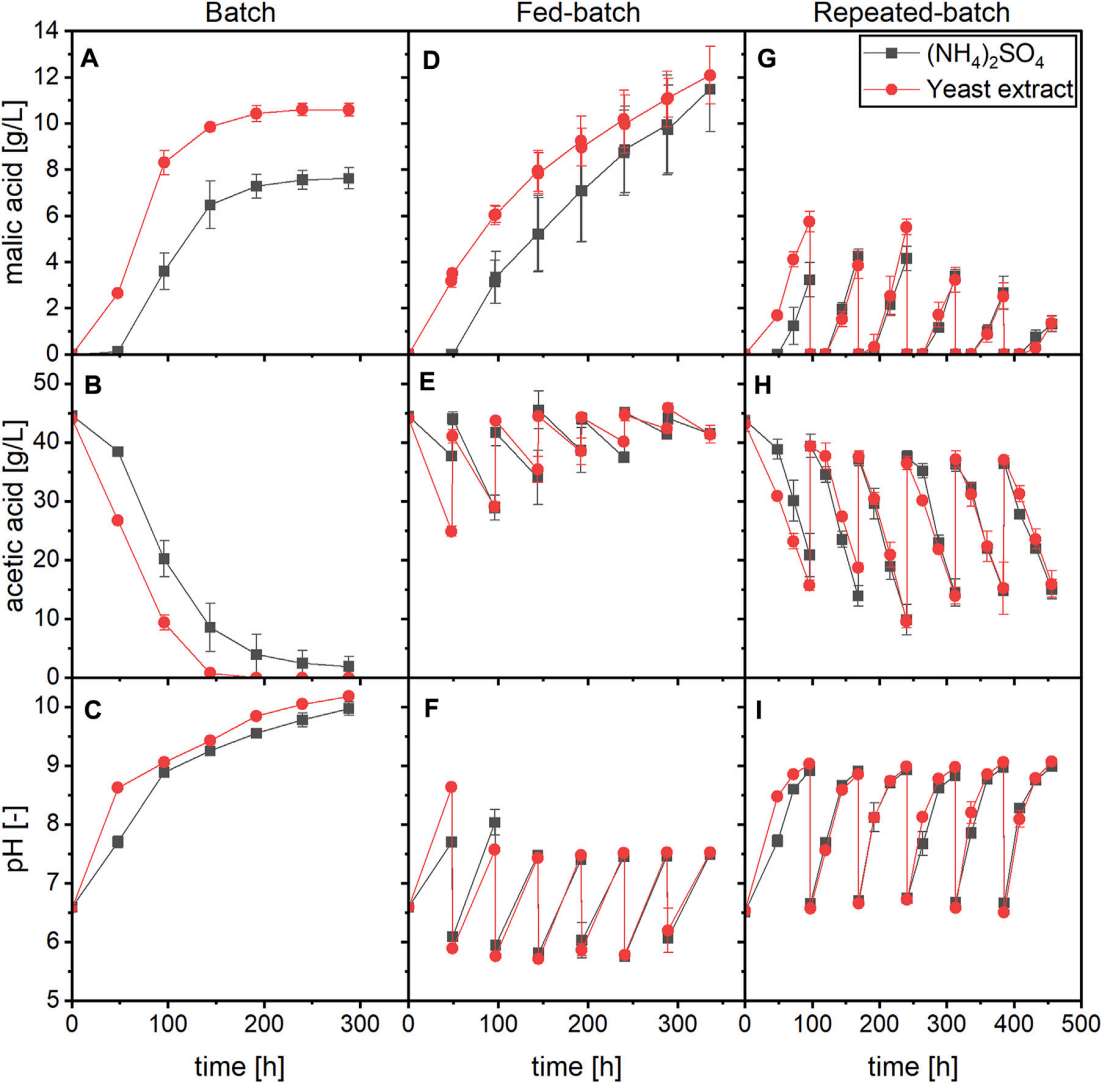
Time course of malic acid production, substrate consumption and pH for a batch **(A–C)**, fed-batch **(D–F)** and repeated-batch **(G–I)** process with *A. oryzae* using (NH_4_)_2_SO_4_ or yeast extract as nitrogen source. Datapoints represent means ± standard deviation, *n* = 3.

Fed-batch and repeated-batch processes with yeast extract or ammonium sulfate as nitrogen source and acetate as carbon source were performed and compared to the respective batch process. The time course of malic acid production, substrate consumption and pH is displayed in Figure 4.

In the fed-batch process, 5 M acetic acid was added every 48 h to restore a substrate concentration of about 45 g/L (Figure 4E). No further medium components were added besides the substrate. With both nitrogen sources, the period of malic acid accumulation was extended compared to the batch process as production was observed until the end of the cultivation at 336 h. The final malic acid concentrations were therefore higher than in the batch process, amounting to 11.49 ± 1.84 g/L with (NH_4_)_2_SO_4_ and 12.08 ± 1.25 g/L with yeast extract (Figure 4D). However, the substrate consumption and acid production rates decreased in later stages of the fermentation despite adequate carbon availability (Supplementary Figure S4). Through the repeated addition of acetic acid, the pH was prevented from surpassing a value of 9.0 and was mostly below 7.5 except for the first 96 h of fermentation (Figure 4F). Immediately after addition of the acid, pH values between 5.5 and 6.0 were measured. In growth experiments performed with *A. oryzae*, the germination of conidia was inhibited at a pH of 6.0 with 45 g/L acetic acid as substrate (Supplementary Figure S5). It is therefore possible that the low pH obtained directly after feeding negatively affected *A. oryzae* although further acid production was not prevented.

Malic acid productivity also declined in later stages of the repeated-batch process. In these experiments, the medium was completely exchanged after the first 96 h and then every further 72 h. Through the repeated medium exchange, the pH was kept between values of about 6.5 and 9.0 (Figure 4I). In the first batch, 3.23 ± 0.74 g/L malic acid was obtained in cultures with (NH_4_)_2_SO_4_ compared to 5.74 ± 0.45 g/L with yeast extract. Starting with the fourth medium exchange, malic acid production declined for both nitrogen sources while the substrate consumption remained similar (Figures 4G,H). In the sixth and last batch, 1.30 ± 0.33 or 1.34 ± 0.34 g/L malic acid were measured with ammonium sulfate or yeast extract, respectively. Interestingly, a difference between the two nitrogen sources was observed mainly in the first batch, while both substrate consumption and malic acid production were mostly similar in later cycles. A lag phase occurred after each medium exchange, which is illustrated by the absence or very low production of malate during the first 24 h of cycles No. 2–6, and a lower acetate consumption rate than between the following two samplings (Supplementary Figure S6). The ammonium concentration was monitored for the cultures containing (NH_4_)_2_SO_4_, and the nitrogen source was consumed during the first 24 h of each cycle, indicating biomass formation (Supplementary Figure S7). Ammonium was only depleted at the end of each batch phase, meaning that nitrogen was not limiting until later stages in the cycles. Since each batch started with a new growth phase, cultures with yeast extract were expected to exhibit a shorter lag phase not only in the first but also in later cycles, but this was not observed.

Table 4 summarizes the calculated values of the three process modes for a cultivation time of 240 h, which comprises the first four feeds of the fed-batch and the first three medium changes of the repeated-batch process. For the batch process, the values in parentheses represent calculations performed for a cultivation time of 144 h, since the acid production was nearly completed within this period. As already displayed in the previous chapter, the batch process with yeast extract performed better than that with (NH_4_)_2_SO_4_, resulting in malic acid yields of 0.24 ± 0.01 and 0.18 ± 0.00 g/g, respectively. Regarding the fed-batch process, the yield for cultures with (NH_4_)_2_SO_4_ was slightly higher than in the batch process (0.20 ± 0.02 g/g) and slightly lower in the cultures with yeast extract (0.21 ± 0.02 g/g). The lower yield of the fed-batch process with yeast extract could be due to a higher biomass accumulation in the beginning of the main culture, as demonstrated in the growth experiments, which then required more carbon for maintenance metabolism throughout the fermentation. The amount of malic acid produced within 240 h was highest in the repeated-batch process with 1.28 ± 0.09 g ((NH_4_)_2_SO_4_) and 1.58 ± 0.25 g (yeast extract). The yield of the repeated-batch processes, however, was lowest. The productivities of the repeated-batch process after 240 h were similar to those of the batch process considering a cultivation time of 144 h. Thus, a high productivity could be maintained at least in the first three cycles of the repeated-batch process.

**TABLE 4:** Fermentation results for cultivations of *A. oryzae* using different process modes after 240 h. Values are means ± standard deviation, *n* = 3.

Process mode	Nitrogen source	Substrate metabolized [g]	Malic acid [g]	Y_P/S, malic acid_ [g/g]^b^	Malic acid productivity [g/L/h]	Total acid [g]^c^	Y_P/S, total acid_ [g/g]	Total acid productivity [g/L/h]
Batch^a^	(NH_4_)_2_SO_4_	4.63 ± 0.22 (3.96 ± 0.42)	0.83 ± 0.04 (0.71 ± 0.11)	0.18 ± 0.00 (0.18 ± 0.01)	0.031 ± 0.002 (0.045 ± 0.007)	1.52 ± 0.11 (1.36 ± 019)	0.33 ± 0.01 (0.34 ± 0.01)	0.058 ± 0.004 (0.086 ± 0.012)
	Yeast extract	4.84 ± 0.01 (4.76 ± 0.06)	1.17 ± 0.03 (1.08 ± 0.02)	0.24 ± 0.01 (0.23 ± 0.00)	0.044 ± 0.001 (0.068 ± 0.001)	2.06 ± 0.03 (1.97 ± 0.03)	0.42 ± 0.01 (0.41 ± 0.01)	0.078 ± 0.001 (0.124 ± 0.002)
Fed-batch	(NH_4_)_2_SO_4_	5.02 ± 0.51	1.01 ± 0.21	0.20 ± 0.02	0.036 ± 0.008	2.03 ± 0.40	0.40 ± 0.06	0.067 ± 0.013
	Yeast extract	5.83 ± 0.63	1.21 ± 0.18	0.21 ± 0.02	0.042 ± 0.005	2.07 ± 0.53	0.38 ± 0.03	0.071 ± 0.017
Repeated-batch	(NH_4_)_2_SO_4_	7.80 ± 0.50	1.28 ± 0.09	0.16 ± 0.00	0.048 ± 0.003	2.43 ± 0.20	0.31 ± 0.01	0.098 ± 0.008
	Yeast extract	7.71 ± 0.52	1.58 ± 0.25	0.20 ± 0.02	0.060 ± 0.010	2.91 ± 0.42	0.38 ± 0.03	0.116 ± 0.017

^a^ Values in parentheses show the results after 144 h of cultivation, during which most of the product formation was detected in batch processes.

^b^ Y_P/S, malic acid_ [g/g] = substrate specific malic acid yield calculated as g (malic acid)/g (consumed acetic acid).

^c^ Refers to all organic acids quantified (see Supplementary Table S3).

The total acid composition after 240 h was largely similar between the process modes with a malic acid ratio of 52%–57% and succinic acid representing 35%–40% (Supplementary Table S3).

## Discussion

### Effect of temperature on organic acid production

This study evaluated the effect of four cultivation temperatures in the range of 29–38°C on organic acid production with the carbon sources acetate and glucose. Generally, the accumulation of organic acids was accelerated with increasing temperature and total acid concentration was highest in cultures incubated at 38°C. However, the ratio of malic acid to by-products was best at 32°C.

The difference in percentages of the main products malate and succinate between the two carbon sources can be explained by the different pathways for substrate metabolization and L-malic acid production. With glucose, the reductive tricarboxylic acid (rTCA) cycle taking place in the cytosol was identified as main pathway for malate synthesis in *Aspergillus flavus* (Peleg et al., 1989). In this pathway, pyruvate carboxylase first catalyzes the carboxylation of pyruvate to oxaloacetate while fixing CO_2_. Then, malate dehydrogenase reduces oxaloacetate to malate. The theoretical malate yield of this pathway from glucose is 2 mol/mol. With acetate, on the other hand, malate is probably mainly produced through the glyoxylate cycle which reduces the maximum theoretical yield to 0.5 mol/mol considering only the carbon balance. This bypass of the citric acid cycle involves the enzymes isocitrate lyase which converts isocitrate to glyoxylate and succinate, and malate synthase which uses glyoxylate and acetyl-CoA for the synthesis of malate (Armitt et al., 1970; Armitt et al., 1971; Ries et al., 2021). Thus, with the carbon source acetate, the production of one mole of malate is accompanied by the synthesis of one mole of succinate.

The malic acid concentration obtained in cultures with acetate incubated at 32°C was only about 21% of that obtained with glucose after 144 h. In these cultivations, initial substrate concentrations were used which are typical for processes with the respective carbon source. In fermentations based on glucose, a much higher initial carbon concentration can be applied and substrate titers of 100 g/L and above are commonly used for efficient malic acid production (Dai et al., 2018). With acetate, however, substrate inhibition is observed at much lower concentrations and the utilization of 55 g/L acetic acid already resulted in a reduced production rate (Kövilein et al., 2021). The inhibiting effect of acetate is caused by the presence of its protonated form, since acetic acid can enter the cytoplasm through diffusion, then dissociates and thereby leads to an intracellular acidification (Stratford et al., 2009). High proton concentrations can impede the proton motive force and thus inhibit ATP synthesis. Furthermore, the increased concentration of acetate ions can contribute to osmotic stress (Kiefer et al., 2021).

The temperature effect was especially pronounced regarding the ratio between malate and succinate in cultures with acetate, as succinate became the main product at the highest temperature tested. An increased succinate concentration at higher temperatures may indicate an elevated activity of enzymes involved in the production of succinic acid, or an inhibition of enzymes involved in its further metabolization. It is also conceivable that the temperature affects the transport of the compounds. However, this cannot be conclusively determined until the effects of temperature on individual enzymes of *A. oryzae* are studied. For *Aspergillus kawachii* (now *A. luchuensis*), for example, down-regulation of fumarase and up-regulation of malate synthase was observed when the temperature was lowered from 40 to 30°C. Furthermore, a higher citric acid concentration was obtained during cultivation of the same strain at 30°C compared to 40°C, which is consistent with the results obtained with glucose in the current study (Futagami et al., 2015).

Finding the process-specific ideal temperature is thus of great importance for optimizing product yields which was also demonstrated by other authors. For succinic acid production with *A. niger*, a temperature of 35°C was found optimum (Yang et al., 2020), while 33°C was best for itaconic acid production with *A. terreus* (Saha et al., 2019). Regarding malic acid production with an engineered strain of *A. oryzae* FMME 338, a slightly higher titer was quantified in cultures incubated at 30°C compared to 32 and 34°C, while less glucose was consumed (Chen et al., 2019). In these studies, the temperature resulting in the highest product concentration differed from the one yielding the highest biomass concentration. Therefore, it might be interesting to evaluate the effect of a temperature shift between the initial growth phase and the production phase in the main culture. The reported studies did not investigate the effect of cultivation temperature on the production of possible side products. Regarding the by-product spectrum in the current study, a temperature of 32°C was most suitable for malic acid production with both glucose and acetate.

### Effect of nitrogen source on growth and L-malic acid production

In this study, several nitrogen sources were evaluated both for growth and organic acid production. With the defined nitrogen sources (NH_4_)_2_SO_4_, urea and glutamate, similar biomass concentrations were obtained. With glutamate, however, the biomass yield related to the consumed acetate was higher compared to cultures containing (NH_4_)_2_SO_4_ and urea. This was also observed during organic acid production and is probably due to glutamate not only functioning as nitrogen but also as carbon source. Although urea theoretically provides one carbon atom per molecule, it does not appear to be utilized as carbon source since no considerable effect on biomass yield was observed. Urea is likely metabolized by urease in *A. oryzae*, converting urea to CO_2_ and ammonia (Strope et al., 2011).

The lowest biomass concentration and yield was determined with sodium nitrate. Like urea, this nitrogen source is metabolized *via* ammonium in *Aspergillus* species. First, NO_3_^−^ is reduced to NO_2_^−^ by nitrate reductase followed by a further reduction to NH_4_^+^ by nitrite reductase (Amaar and Moore, 1998; Schinko et al., 2010). This process is energy-consuming which could explain the lower biomass concentration and delayed germination.

Key enzymes involved in ammonium assimilation are glutamate dehydrogenase, glutamine synthetase and glutamate synthase. For *Aspergillus* species it is assumed that the pathway *via* glutamate dehydrogenase is the main pathway for the metabolization of ammonium and therefore also urea and nitrate. This was demonstrated by the observation that *gdhA*-lacking mutants of *A. nidulans* displayed lagged or poor growth when ammonium, nitrate or urea was used as nitrogen source (Arst and MacDonald, 1973; Kinghorn and Pateman, 1973). The NADP-dependent glutamate dehydrogenase (encoded by *gdhA*) catalyzes the amination of α-ketoglutarate, yielding glutamate which is then further used in anabolic reactions. The NAD-linked glutamate dehydrogenase (*gdhB*), however, is assumed to mainly be involved in glutamate catabolism, hence the deamination of glutamate, as mutants deficient in this enzyme did not grow on glutamate as the sole carbon source (Kinghorn and Pateman, 1973). The observation that growth of a double mutant deficient in both glutamate dehydrogenase genes on ammonium, nitrate and urea was delayed but not completely inhibited, suggested another pathway to be involved in ammonium assimilation (Kinghorn and Pateman, 1976). Glutamine synthetase uses ammonia to aminate glutamate, forming glutamine. Together with α-ketoglutarate, the glutamine is then used by glutamate synthase to form two molecules of glutamate. The involvement of glutamate synthase (*gltA*) in the nitrogen metabolism of *A. nidulans* was demonstrated by observing the growth of mutants deficient in this enzyme. When both *gdhA* and *gltA* were lacking, no growth was observed with the nitrogen sources ammonium and nitrate, whereas biomass was formed with glutamate or glutamine (Macheda et al., 1999). As urea and nitrate first need to be converted to ammonia and then glutamate, it is generally assumed that the latter two are preferred nitrogen sources. This was confirmed for NaNO_3_ in the growth experiments and urea during organic acid production presented here.

The utilization of complex nitrogen sources considerably increased biomass production. Besides nitrogen, these compounds also provide carbohydrates, amino acids, vitamins and trace elements (Klotz et al., 2017). The advantage of trace element availability during growth was demonstrated by an increase in dry biomass concentration with the addition of Hutner’s trace element solution. The trace element supplementation was sufficient to enhance biomass production to levels similar to those obtained with the complex nitrogen sources. Different than the trace element solution, however, the complex nitrogen sources also caused an earlier onset of germination. The enhanced biomass production and earlier germination could have been caused by the availability of carbohydrates or the supply of free amino acids as direct precursors for protein synthesis provided by the complex nitrogen sources. For *A. niger*, some amino acids were found to trigger germination as opposed to other nitrogen sources such as sodium nitrate, urea, or ammonium sulfate (Hayer et al., 2014). In addition, phosphate availability, which was likely increased in cultures with complex nitrogen sources, may have played a role. Regarding the organic acid production in the main culture, especially yeast extract had a positive influence as it increased the productivity in the beginning of the fermentation. The composition and therefore the effect of complex nitrogen sources strongly depend on their manufacturing process. The type or brand can therefore have a significant impact on growth and production (Sørensen and Sondergaard, 2014; Klotz et al., 2017). A detailed analysis of the nitrogen source’s composition is thus necessary for determining the effects conclusively.

The concentration of the nitrogen sources applied in the media was determined according to their total nitrogen concentration, with the aim of keeping it at 0.85 g/L in the pre-culture and 0.25 g/L in the main culture. This resulted in rather high concentrations of the complex nitrogen sources especially in the pre-culture. Further experiments should evaluate the minimum concentration of these compounds required to obtain a positive effect on growth or malic acid production. It would also be interesting to study malic acid production in media with (NH_4_)_2_SO_4_ as the main nitrogen source, supplemented with low concentrations of yeast extract.

Using glucose, several nitrogen sources have already been evaluated for malic acid production. With *A. oryzae* NRRL3488 the utilization of peptone resulted in a 34% higher malic acid productivity during the stationary phase compared to cultures with ammonium sulfate, yielding a final titer of 30.27 ± 10.5 g/L compared to 22.27 ± 0.46 g/L, respectively. Given that the yields were similar with the two nitrogen sources, the authors suggested a difference in biomass production as a reason for this observation (Knuf et al., 2013). Ding et al. compared malic acid production with *A. oryzae* CCTCC M 2016401 using different ammonium compounds as well as urea and tryptone. Among the defined nitrogen sources, they found the highest malic acid titer of 30.7 ± 0.9 g/L with (NH_4_)_2_SO_4_ but a significantly higher concentration of 70.2 ± 0.7 with tryptone (Ding et al., 2018). In the current study, an enhanced organic acid production was mainly observed in the beginning of the fermentation with the complex nitrogen sources but smaller differences towards the end. This is likely due to the depletion of acetate which prevented further acid production. Ding et al. (2018) found optimizing both the C/N ratio as well as the absolute concentrations of both the carbon and nitrogen source effective for enhancing the L-malate titer. The C/N ratio was also identified as an important parameter for cultivation of *A. oryzae* DSM 1863 with glucose, as the concentration of the side product fumaric acid was greatly affected and ranged from 0.70 ± 0.07 g/L (C/N of 100:1) to 8.44 ± 1.55 g/L (C/N of 300:1) while the malic acid concentration was about 52–55 g/L (Ochsenreither et al., 2014). Therefore, it may be interesting to investigate the effects of different C/N ratios on the organic acid spectrum produced from acetate in future studies.

### Comparison of process modes for L-malic acid production

Substrate inhibition is a disadvantage of batch processes which is especially relevant using acetate as carbon source. Malic acid production with *A. oryzae* was already inhibited at concentrations above 50 g/L acetic acid, resulting in a maximum product concentration of less than 10 g/L in batch mode (Kövilein et al., 2021). Therefore, a fed-batch and repeated-batch process was evaluated aiming to enhance product synthesis. Given that yeast extract accelerated malic acid production, all experiments were run with this nitrogen source in addition to cultures with ammonium sulfate.

Whereas the production rate in the batch process with both nitrogen sources considerably decreased after 144 h of cultivation due to substrate depletion or high pH values, the period of malic acid production was prolonged in both fed-batch and repeated-batch mode. In the fed-batch process, furthermore, higher maximum malic acid concentrations were obtained. However, the productivity decreased during the cultivation. This resulted in similar volumetric production rates for the fed-batch and batch processes considering a cultivation time of 240 h, and even decreased production rates for the fed-batch when comparing it to the first 144 h of the batch process. A different observation was made in fed-batch cultivations for L-malic acid production with a modified strain of *A. oryzae* performed by Liu et al. (2017) They reported a 13% increase in productivity in the fed-batch process (1.38 g/L/h) compared to the batch process (1.22 g/L/h) with glucose as substrate. A reason for the decrease in productivity observed in the current study could be the inhibitory effect of acetic acid which is pH dependent. The pH of the fermentation medium was temporarily brought to values between 5.5 and 6.0 by the addition of acetic acid. As discussed above, the presence of acetic acid is responsible for the inhibiting effect of acetate. Acetic acid is a weak organic acid with a pKa of 4.75 and therefore almost completely dissociated at physiological pH values commonly found in fermentation media. However, the lower the pH of the medium, the more acetate is present as acetic acid. Although the low pH values reached due to the feeding did not prevent further malate production, repeated exposure to increased concentrations of the free acid could have had an adverse effect on *A. oryzae* over time. In experiments evaluating the effect of pH on growth, no biomass formation or germination was found at a pH of 6.0 when 45 g/L acetic acid was used as substrate, demonstrating a negative effect of the pH range temporarily reached during the fed-batch. In experiments testing a substrate range of 10–55 g/L acetic acid in batch mode, 45 g/L was identified as optimal initial concentration whereas low acetate concentrations resulted in an increased oxalic acid production (Kövilein et al., 2021). The feeding strategy applied in this study thus aimed to maintain a rather high substrate level. Therefore, a concentration of about 45 g/L acetic acid was restored every 48 h although the carbon source was not depleted. The gradual decrease in substrate consumption suggests that this feeding strategy needs to be optimized regarding the target substrate concentration in combination with the pH value. Further experiments should therefore investigate whether maintaining a constant pH and substrate concentration in the controlled environment of a bioreactor is beneficial for fed-batch malic acid production. Another reason for the decline in productivity during the fed-batch process might be the gradual dissolution of the CaCO_3_ through the repeated feeds of acetic acid. A major function of CaCO_3_ during malic acid production with glucose is the buffering of the pH (Schmitt et al., 2021). Moreover, CaCO_3_ could supply CO_2_ required by pyruvate carboxylase, or have a positive effect on malate production by providing calcium ions which can form poorly soluble calcium malate (Peleg et al., 1989; Zambanini et al., 2016). Although the function of CaCO_3_ for malic acid production from acetate with *A. oryzae* has yet to be determined conclusively, elevated concentrations seem to be favorable (Kövilein et al., 2021). The addition of CaCO_3_ during a fed-batch process could therefore be considered in future research.

In the repeated-batch process, the volumetric productivity was maintained in the first three cycles, but then gradually decreased from the fourth cycle onwards. A productivity decline in later cycles during repeated-batch or repeated fed-batch cultivation was also reported for other processes such as l-tyrosine production with *E. coli* (Li et al., 2020), lipid production with *Rhodosporidium toruloides* (Zhao et al., 2011), kojic acid production with *A. oryzae* (Wan et al., 2005), or L-malic acid production with *A. oryzae* and glucose as carbon source (Schmitt et al., 2021). The authors indicated a decrease in cell viability, the accumulation of undesired by-products or temporary substrate limitation as possible explanations. Yu et al. (2018) reported a decrease in productivity with increasing batch number during citric acid production with *A. niger* when free cells were used, while a high productivity was maintained also at later process stages with mycelium immobilized on a porous foam. Although the productivity declined in the results presented here, the substrate consumption remained comparable, resulting in lower yields. This is most likely attributed to an increased biomass concentration, although the biomass content was not determined. In each cycle, the medium was completely replaced with medium containing 0.25 g/L nitrogen. Within the first 24 h after each medium replacement no or very little malate production was detected but acetate and ammonium were consumed. The nitrogen consumption was measured for the cultures with (NH_4_)_2_SO_4_ and a depletion of nitrogen was only observed between 48 and 72 h in batches 2–6. As long as nitrogen was available, some carbon was most likely used for the formation of new biomass instead of malic acid production. By reducing or omitting the nitrogen source in the replacement medium, it may be possible to direct more acetate towards malate production, which could allow for higher productivities in later batches. However, Schmitt et al. (2021) showed that in repeated-batch experiments with *A. oryzae* and glucose as carbon source, the complete omission of the nitrogen source in the replacement medium caused a gradual reduction of malic acid production between cycles. Since malic acid production based on acetate differs from the process using glucose in aspects such as the metabolic pathway towards malate formation or the morphology (Kövilein et al., 2021), evaluating the effect of nitrogen availability in repeated-batch experiments with acetate could be considered. The lag phase observed in each cycle of the repeated-batch experiments could also be associated with stress due to the pH drop from values of about 9.0 to 6.5 caused by the medium exchange, or the filtration and washing of the biomass between the cycles. Although this approach was successful in removing all products and allowed for the reutilization of the entire amount of biomass, it might be better to replace the fermentation medium only partly. During itaconic acid production with *Ustilago cynodontis* in a repeated-batch process, a lag phase was also observed which the authors attributed to the centrifugation step performed between the cycles to recover the biomass (Hosseinpour Tehrani et al., 2019). Determining the optimum replacement ratio can be beneficial for repeated-batch processes. Regarding repeated fed-batch production of cellulase with *Penicillium oxalicum*, for example, a replacement percentage of 50% resulted in the highest volumetric productivity after 144 h (Han et al., 2017). Furthermore, during isocitric acid production with *Yarrowia lipolytica*, a medium removal scheme replacing 20%–80% of the medium after 24–72 h resulted in high product titers even after more than 700 h of cultivation (Morgunov et al., 2019).

In summary, higher maximum malate titers were achieved in the fed-batch process, and the repeated-batch process allowed to maintain the productivity at least in the first three cycles. However, neither the fed-batch nor the repeated-batch process showed clearly positive results regarding yield and volumetric productivity when compared to the 144 h-values of the batch process. This might be owed to the limitations of the shake flask environment in which it is difficult to control the pH. To prevent the productivity from declining in later fermentation stages in the fed-batch and repeated-batch process, factors like the nutrient availability, the strategy for biomass separation or the replacement ratio likely need to be optimized.

## Conclusion

In this study, several aspects of L-malic acid production were evaluated with the focus on acetate as carbon source. The cultivation temperature was identified as an important process parameter for controlling the side product spectrum. With increasing temperature, malic acid production accelerated but at the same time, the proportion of succinic acid increased. The highest share of malic acid from acetate was quantified at 32°C, which was also found in comparative experiments performed with glucose. The choice of nitrogen source largely affected fungal growth and the highest biomass concentrations were obtained with yeast extract or peptone. Possibly, trace elements provided by the complex nitrogen sources contributed to this effect, since the utilization of ammonium sulfate together with a trace element solution resulted in similar biomass concentrations. Regarding malic acid synthesis, the utilization of yeast extract proved to be particularly advantageous, as production was enhanced at the beginning of cultivation. Since organic acid production with acetate is limited due to substrate inhibition in a batch process, fed-batch and repeated-batch strategies were evaluated. Although the maximum malic acid concentration was enhanced and the production period was extended compared to the batch process, optimization is required to increase yields and maintain high productivities.

## Data Availability

The original contributions presented in the study are included in the article/Supplementary Material, further inquiries can be directed to the corresponding author.
