# Supplementary material for: Effect of process mode, nitrogen source and temperature on L-malic acid production with Aspergillus oryzae DSM 1863 using acetate as carbon source
Source: Front Bioeng Biotechnol. 2022 Oct 14;10:1033777. doi: 10.3389/fbioe.2022.1033777 (PMC9614319; doi:10.3389/fbioe.2022.1033777)
Supplement: Supplementary file 1 [file DataSheet1.PDF]

Supplementary Material

Table S1. Organic acid distribution for cultivations of *A. oryzae* at different temperatures after 144 h.

Substrate	T [°C]	Organic acids [%]						
		Malate	Succinate	Fumarate	Pyruvate	α-Keto-glutarate	Oxalate	Citrate
**Acetate**	29	52.5 ± 2.4	34.8 ± 2.3	4.5 ± 0.4	0.6 ± 0.1	0.2 ± 0.1	6.2 ± 1.6	1.2 ± 0.6
	32	53.7 ± 1.6	38.7 ± 1.4	3.8 ± 0.4	0.7 ± 0.0	0.3 ± 0.1	2.2 ± 1.9	0.6 ± 0.2
	35	48.6 ± 0.9	43.9 ± 0.6	4.0 ± 0.1	1.0 ± 0.1	0.3 ± 0.1	1.2 ± 0.1	1.0 ± 0.4
	38	43.3 ± 1.1	51.5 ± 1.0	2.8 ± 0.1	1.1 ± 0.0	0.4 ± 0.1	0.3 ± 0.1	0.8 ± 0.2
**Glucose**	29	64.7 ± 2.4	16.5 ± 1.4	2.5 ± 0.0	0	0.2 ± 0.0	1.7 ± 0.1	14.4 ± 1.7
	32	73.8 ± 1.0	16.7 ± 0.2	1.5 ± 0.1	0.8 ± 0.2	0.4 ± 0.0	0.2 ± 0.1	6.6 ± 0.9
	35	68.8 ± 2.1	18.8 ± 1.3	1.4 ± 0.1	4.0 ± 0.4	0.9 ± 0.1	0.1 ± 0.0	6.1 ± 0.5
	38	63.4 ± 2.0	21.3 ± 1.4	1.5 ± 0.2	7.3 ± 0.5	1.7 ± 0.1	0.1 ± 0.0	4.7 ± 0.2

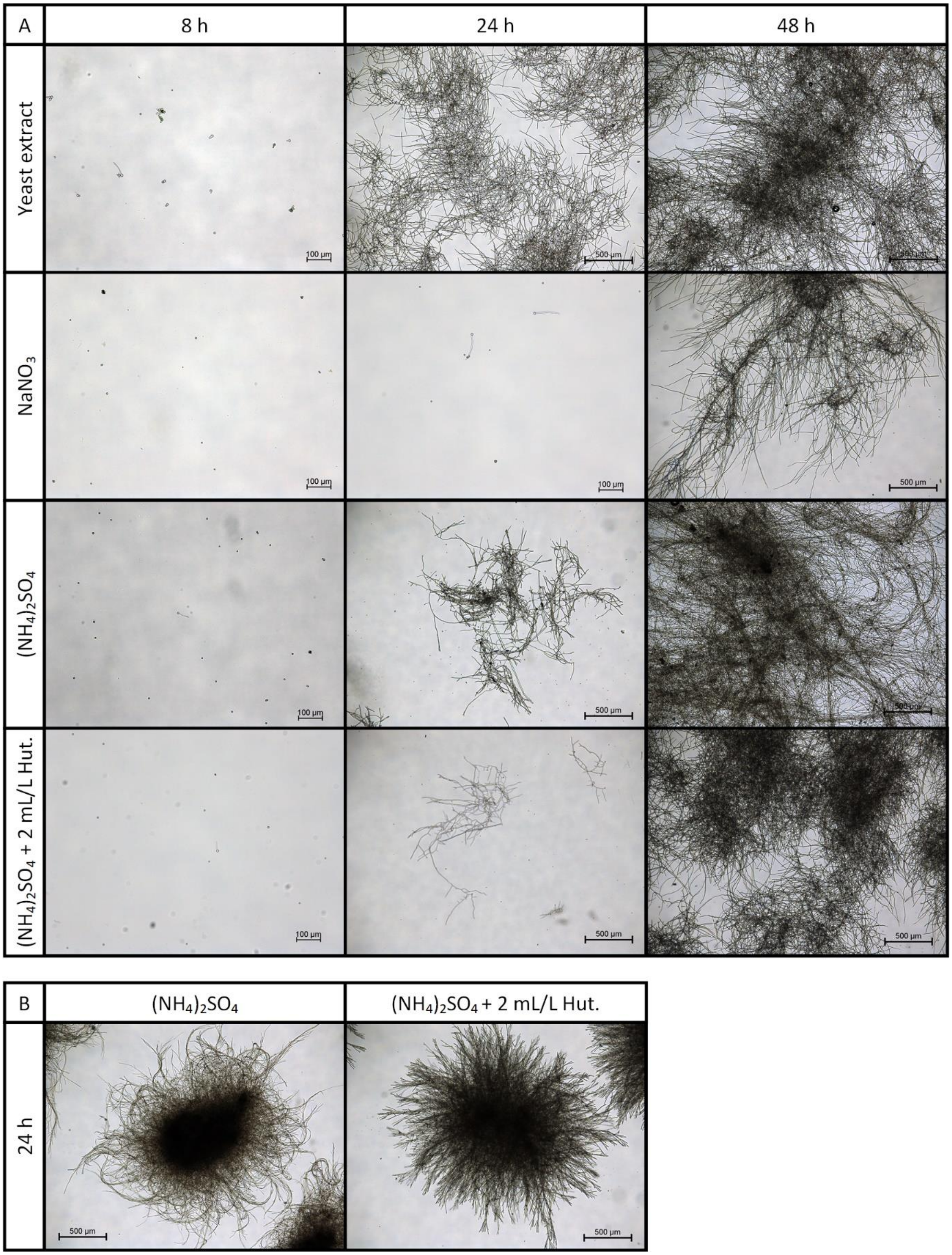

Figure S1. Morphology of *A. oryzae* after growth with different nitrogen sources and Hutner's trace element solution using acetate (A) or glucose (B) as carbon source.

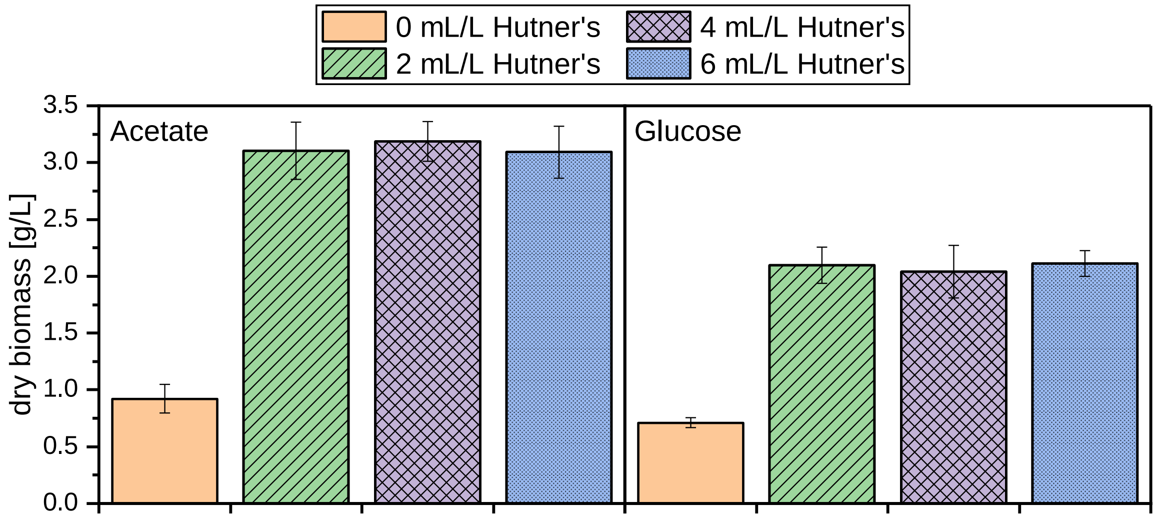

Figure S2. Dry biomass concentration after 48 h (acetate) or 24 h (glucose) of growth in pre-culture medium with different concentrations of Hutner's trace element solution. Datapoints represent means ± standard deviation, n = 3.

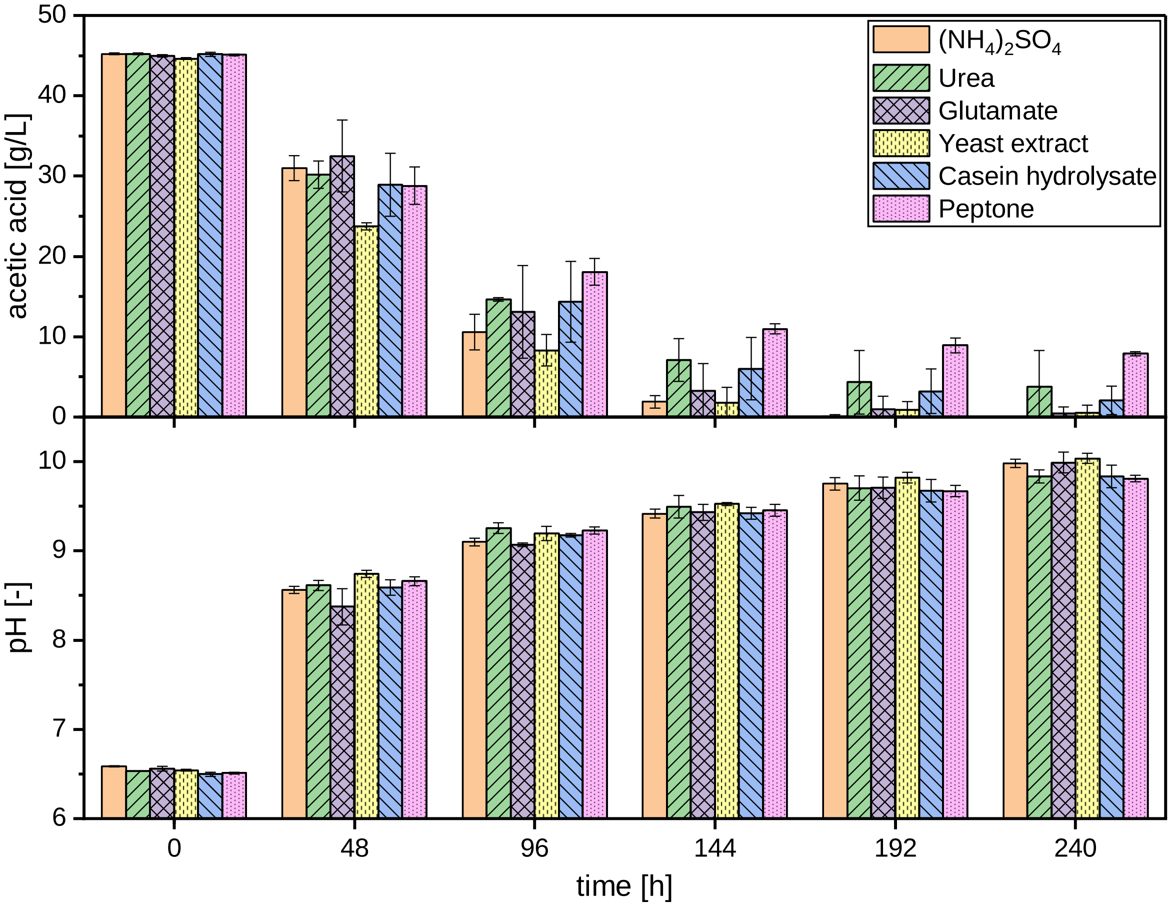

Figure S3. Time course of acetic acid concentration and pH during cultivations of *A. oryzae* with different nitrogen sources. Datapoints represent means ± standard deviation, n = 3.

Table S2. Organic acid distribution for cultivations of *A. oryzae* with different nitrogen sources after 144 h.

**Nitrogen source**	**Organic acids [%]**						
	**Malate**	**Succinate**	**Fumarate**	**Pyruvate**	**α-Keto-glutarate**	**Oxalate**	**Citrate**
**$(NH_4)_2SO_4$**	54.8 ± 2.3	37.1 ± 2.2	3.7 ± 0.4	0.6 ± 0.0	0.4 ± 0.0	1.8 ± 0.4	1.6 ± 0.5
**Urea**	49.3 ± 1.5	38.1 ± 1.5	5.7 ± 0.5	1.0 ± 0.0	0.6 ± 0.1	4.5 ± 1.2	0.8 ± 0.8
**Glutamate**	53.9 ± 0.8	35.9 ± 1.9	4.1 ± 0.1	0.6 ± 0.0	0.7 ± 0.1	2.9 ± 1.1	1.9 ± 0.4
**Yeast extract**	52.5 ± 1.6	37.1 ± 1.0	4.1 ± 0.4	0.6 ± 0.0	0.5 ± 0.1	3.2 ± 1.3	1.9 ± 0.4
**Casein hydrolysate**	52.0 ± 1.3	36.1 ± 1.4	4.5 ± 0.6	1.0 ± 0.1	0.5 ± 0.0	3.8 ± 2.3	2.1 ± 0.1
**Peptone**	50.7 ± 0.7	38.7 ± 0.7	4.1 ± 0.1	0.7 ± 0.1	0.5 ± 0.1	3.9 ± 1.3	1.4 ± 0.3

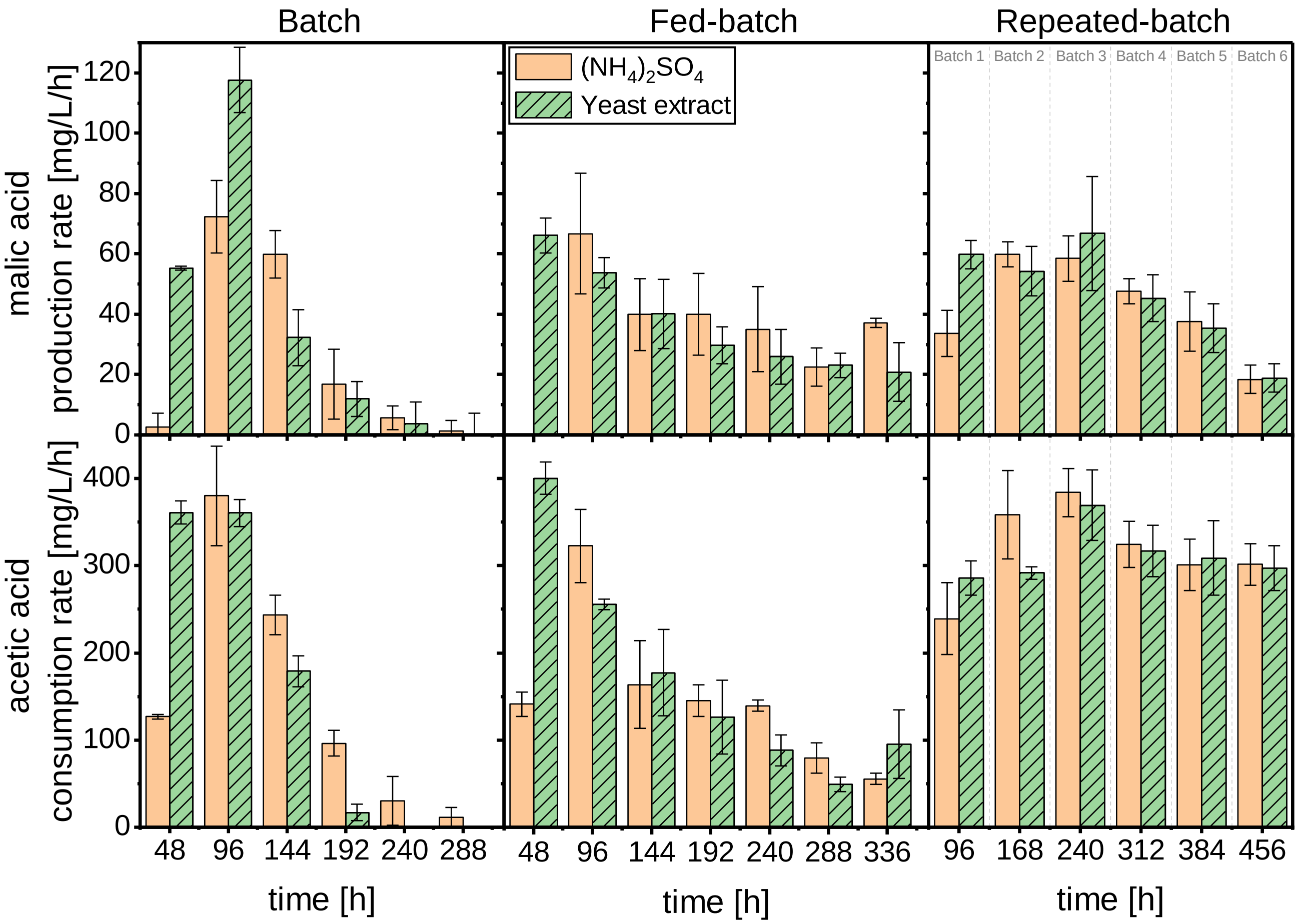

Figure S4. Malic acid production and acetic acid consumption rates for different process modes. Values were calculated between measurements every 48 h for the batch and fed-batch process. For the repeated-batch process, values were calculated for each batch. Datapoints represent means ± standard deviation, n = 3.

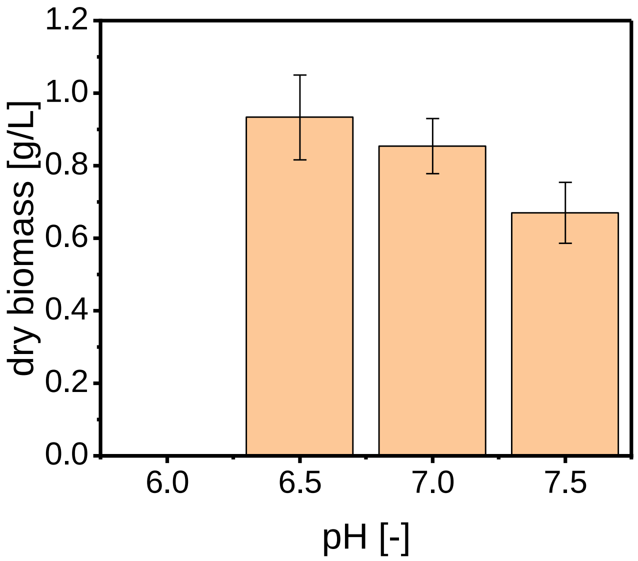

Figure S5. Dry biomass concentration of *A. oryzae* after 48 h of cultivation in medium with 45 g/L acetic acid adjusted to different pH values. Datapoints represent means ± standard deviation, n = 3.

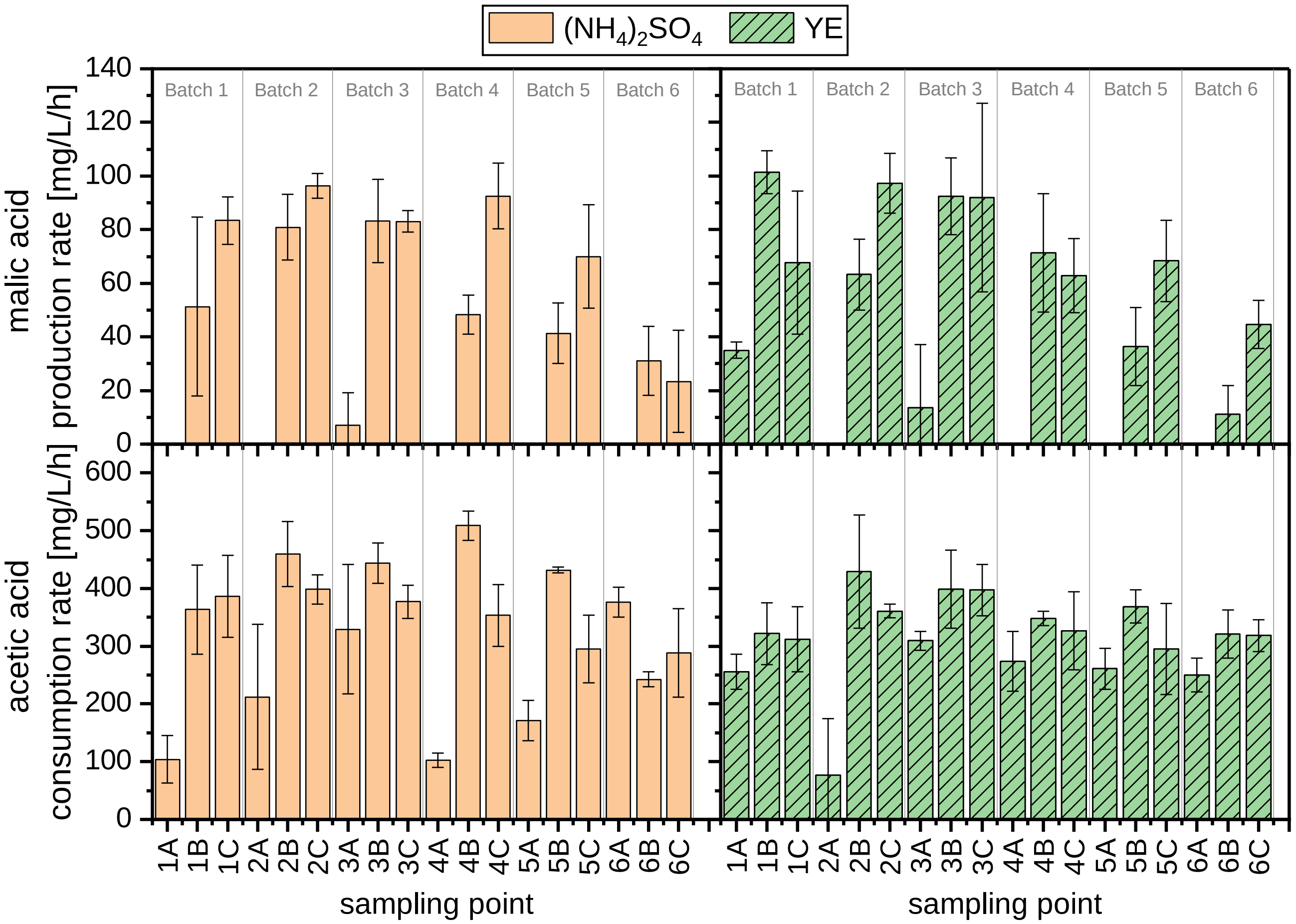

Figure S6. Malic acid production rate and acetic acid consumption rates calculated between each measurement point (see Figure 4) for the repeated-batch process. Hence, "1A" refers to the rates calculated for the first 48 h in the first batch cycle, "1B" are the rates calculated between 48 h and 72 h in the first batch, values for "2A" were determined for the first 24 h of the second batch and so on. Datapoints represent means ± standard deviation, n = 3.

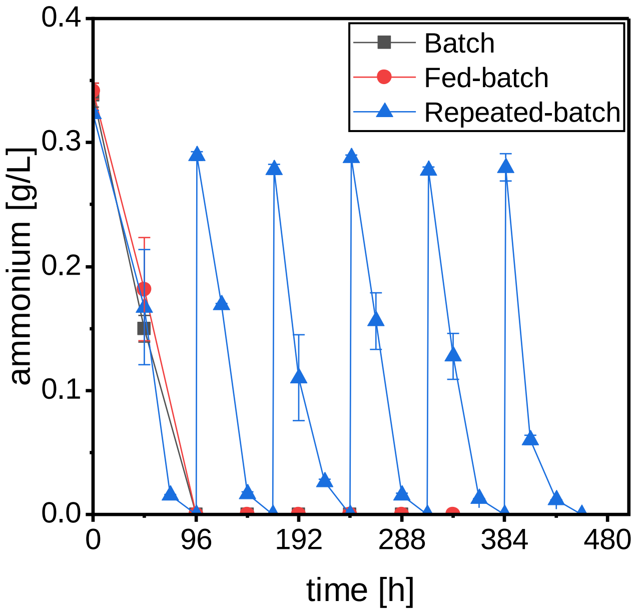

Figure S7. Ammonium concentration for batch, fed-batch and repeated-batch production of L-malic acid with $(NH_4)_2SO_4$ as nitrogen source. Datapoints represent means ± standard deviation, n = 3.

Table S3. Organic acid composition for cultivations of *A. oryzae* using different process modes after 240 h.

Process mode	Nitrogen source	Organic acids [%]						
		**Malate**	**Succinate**	**Fumarate**	**Pyruvate**	**α-Keto-glutarate**	**Oxalate**	**Citrate**
**Batch**[a]	$(NH_4)_2SO_4$	54.6 ± 2.0 (52.3 ± 1.4)	34.9 ± 4.1 (37.5 ± 1.0)	3.9 ± 0.3 (3.9 ± 0.4)	0.6 ± 0.1 (0.6 ± 0.1)	0.4 ± 0.0 (0.3 ± 0.1)	3.7 ± 2.4 (3.5 ± 2.2)	1.9 ± 0.7 (1.9 ± 0.4)
	Yeast extract	56.7 ± 0.8 (55.1 ± 1.2)	35.2 ± 0.3 (37.0 ± 0.7)	3.4 ± 0.1 (3.4 ± 0.0)	0.6 ± 0.1 (0.6 ± 0.1)	0.4 ± 0.0 (0.4 ± 0.0)	1.9 ± 0.5 (1.8 ± 0.4)	1.7 ± 0.1 (1.8 ± 0.3)
**Fed-batch**	$(NH_4)_2SO_4$	54.2 ± 1.0	36.9 ± 1.4	3.1 ± 0.1	0.6 ± 0.1	0.1 ± 0.1	4.0 ± 1.7	1.1 ± 0.2
	Yeast extract	55.2 ± 0.7	39.6 ± 1.0	3.3 ± 0.2	0.6 ± 0.1	0.1 ± 0.1	0.7 ± 0.2	0.5 ± 0.7
**Repeated-batch**	$(NH_4)_2SO_4$	52.5 ± 2.9	36.3 ± 3.7	5.2 ± 0.7	1.0 ± 0.2	0.1 ± 0.1	4.6 ± 1.9	0.4 ± 0.7
	Yeast extract	54.9 ± 3.5	36.5 ± 1.6	4.2 ± 0.5	0.8 ± 0.4	0.3 ± 0.2	2.7 ± 1.6	0.6 ± 0.5

[a] Values in parentheses show the results after 144 h of cultivation, during which most of the product formation was detected in batch processes.
